# Triage and hospitalization outcomes in the geriatric population of an emergency department: A retrospective cohort study comparing the manchester triage system and the emergency severity index

**DOI:** 10.1371/journal.pone.0332304

**Published:** 2025-09-17

**Authors:** Anna Ingielewicz, Marzena Szarafińska, Maciej Zając, Zuzanna Brunka, Mariusz Grażewicz, Mateusz Szczupak, Mariusz Siemiński

**Affiliations:** 1 Department of Emergency Medicine, Faculty of Health Science, Medical University of Gdansk, Gdansk, Poland; 2 Department of Emergency Medicine, Copernicus Hospital, Gdansk, Poland; 3 Department of Emergency Medicine, University of Warmia and Mazury in Olsztyn, Olsztyn, Poland; 4 Department of Anesthesiology and Intensive Care, Copernicus Hospital, Gdansk, Poland; Hospital Sirio-Libanes, BRAZIL

## Abstract

**Introduction:**

Elderly patients in emergency departments (EDs) are at increased risk due to nonspecific symptoms, multimorbidity, and elevated mortality. This study compared the predictive performance of the Manchester Triage System (MTS) and the Emergency Severity Index (ESI) for hospitalization and critical outcomes in geriatric patients and analyzed symptom patterns by age and clinical course.

**Methods:**

This retrospective study included all patients aged ≥18 years admitted to a tertiary ED in northern Poland between January and June 2021. Each patient was concurrently assessed using both MTS and ESI. Data collected included triage level, age group (18–64, 65–79, ≥ 80), sex, mode of arrival, presenting symptoms, and outcomes including hospitalization and ten predefined critical events (e.g., sepsis, admission, urgent surgery). Logistic regression was used to assess associations.

**Results:**

Among 1,063 patients, 475 (44.7%) were aged ≥65. Patients aged 18–64 most commonly presented with abdominal pain or polytrauma, while geriatric patients more frequently reported dyspnea, weakness, and altered mental status. Dyspnea was nearly twice as common in patients ≥80. Weakness (OR = 1.67) and abdominal pain (OR = 1.64) were significantly associated with hospitalization. Hospitalization and critical events were more likely in older adults (OR = 2.03 for ages 65–79; OR = 3.74 for ≥80). In both systems, higher triage urgency was independently associated with greater risk (MTS: OR = 0.51; ESI: OR = 0.43). ESI showed stronger alignment with physiological deterioration and predicted complications such as ICU admission and sepsis more consistently than MTS.

**Conclusions:**

MTS and ESI show limited sensitivity in older patients, particularly with nonspecific presentations. ESI provided better discrimination of clinical urgency. Findings support revising triage systems to account for age, atypical symptoms, and geriatric vulnerability.

## Introduction

With the increasing number of elderly patients seeking care in EDs, accurately identifying those who require urgent attention or hospitalization remains a clinical challenge [1]. Aging is often associated with multiple chronic conditions, polypharmacy, and atypical presentations, such as general weakness, confusion, or nonspecific abdominal pain. These factors complicate triage decisions and may delay timely interventions [2,3].

Two widely used triage systems in Europe and worldwide are the MTS and ESI [4,5]. MTS is symptom-based and utilizes structured algorithms, whereas ESI focuses on acuity and anticipated resource needs. Both systems were originally developed for general ED populations and have limited validation in older patients [6,7].

Numerous studies have highlighted the risk of under-triage in geriatric patients, which can lead to suboptimal outcomes [8,9]. The aim of this study was to compare how MTS and ESI classify elderly patients, and to evaluate how well their classifications align with hospitalization decisions and in-hospital mortality. Understanding the predictive value of these tools in a high-risk demographic may inform potential adjustments or the incorporation of geriatric-specific components. Importantly, under-triage in older adults has been shown to result in delayed recognition of critical illness, increased ICU admissions, and higher short-term mortality, underlying its clinical significance [10]. A recent study compared MTS, ESI, qSOFA, and NEWS in geriatric patients, confirming the vulnerability of this population but focusing primarily on prognostic accuracy across multiple tools [11]. Our study complements and extends this work by providing a detailed head-to-head comparison of MTS and ESI in the same geriatric cohort, with a specific emphasis on hospitalization outcomes and mortality.

## Materials and methods

This was a retrospective cohort study conducted in the ED of a 450-bed multidisciplinary tertiary hospital. The ED serves approximately 110 patients per day and includes designated areas for waiting, resuscitation, and observation/treatment with 14 beds in total. The department is equipped to manage patients of all ages and covers a full spectrum of internal medicine, neurological, and trauma cases.

The study population included all patients who presented to the ED between January 1 and June 30, 2021. Detailed analysis was focused on patients aged 65 years and older. Inclusion was irrespective of referral source—self-presentation, Emergency Medical Services (EMS), or primary care physician referral.

Eligible participants were adult patients with complete medical records. Exclusion criteria were: age below 18 years, incomplete documentation, transfer from the ED to another hospital, admission via fast-track pathways (e.g., acute myocardial infarction or stroke protocols), or death prior to ED admission.

At the time of ED presentation, each patient was evaluated simultaneously using both triage systems: MTS and the ESI. The Manchester Triage System (MTS) is a symptom-oriented tool structured around flowcharts that guide the user through a series of discriminators (e.g., vital signs, intensity of pain, specific clinical features). Based on the first positive discriminator encountered, patients are assigned to one of five urgency categories: level 1 (immediate), level 2 (very urgent), level 3 (urgent), level 4 (standard), and level 5 (non-urgent).

The Emergency Severity Index (ESI) is a five-level triage system that combines initial assessment of acuity with anticipated resource utilization. Patients at level 1 require immediate life-saving interventions, while level 2 indicates high-risk conditions or severe distress. Levels 3–5 are differentiated by the expected number of diagnostic or therapeutic resources needed during the ED visit, with level 3 requiring multiple resources and levels 4–5 fewer or none.

Both systems generate ordinal triage levels that reflect expected urgency; however, MTS relies primarily on presenting symptoms and structured algorithms, whereas ESI explicitly integrates resource prediction in addition to acuity assessment.

Triage was performed by paramedics and nurses with at least five years of experience in the emergency department, all of whom held active certification and formal training in both triage systems. According to hospital protocol, MTS was the primary triage tool; the same healthcare provider concurrently assigned an ESI level, both of which were recorded in the hospital’s medical documentation.

Collected data included age, sex, mode of arrival (self, ambulance/helicopter, or referral), chief presenting complaint, triage levels, and predefined critical outcomes during hospitalization. Data were extracted from the electronic medical records system (Clininet).

Chief complaints or diagnosis upon ED presentation were documented and categorized as: dyspnea, shock, altered mental status, weakness, nausea/vomiting, post-cardiac arrest, edema, headache, chest pain, abdominal pain, limb pain, back pain, isolated trauma, multiple trauma, stroke, peripheral paresis, fever, gastrointestinal bleeding, airway bleeding, gynecological bleeding, and urinary tract bleeding.

Triage performance was evaluated against predefined clinical endpoints indicative of patient severity, including death in the emergency department, in-hospital mortality, sepsis confirmed by positive cultures or procalcitonin level >2 ng/mL, need for urgent hemodynamic intervention, administration of thrombolysis, surgical procedure within 24 hours of ED admission, direct admission to the Intensive Care Unit (ICU) from the ED, ICU admission during hospitalization, blood transfusion within 24 hours of arrival, vasopressor use in the ED, and initiation of mechanical ventilation in the ED. The primary outcome is defined in terms of the occurrence of critical incidents.

Statistical analyses were conducted using Python (pandas, statsmodels, seaborn libraries) and Microsoft Excel. Descriptive statistics were presented as absolute numbers (n) and percentages (%) for categorical variables, and as medians, standard deviations (SD), and interquartile ranges (IQR) for continuous variables.

Group comparisons for categorical variables (e.g., sex, presenting symptoms, mode of arrival, hospitalization, triage level) were performed using the Chi-square (χ²) test or Fisher’s exact test, as appropriate based on cell counts. For continuous variables (e.g., age), the Shapiro–Wilk test was used to assess normality, and the Mann–Whitney U test was used for non-parametric comparisons between groups.

Multivariable logistic regression models were applied to assess independent predictors of hospitalization and critical events. Predictor variables included age group (18–64, 65–79, ≥ 80 years), presenting symptoms (e.g., weakness, abdominal pain), triage level (MTS or ESI), sex, and mode of ED arrival (ambulance/helicopter vs. self-referral). Odds ratios (OR) with 95% confidence intervals (CI) were reported. A p-value < 0.05 was considered statistically significant.

Visualizations included heatmaps for symptom–age–hospitalization relationships, bar plots comparing triage and arrival modes, and forest plots displaying adjusted ORs for critical event prediction by triage level and age group.

This study was a retrospective analysis of medical records and was conducted in accordance with the ethical standards of the hospital and the 1964 Declaration of Helsinki and its later amendments. All data were fully anonymized before being accessed by the researchers. The data for research purposes were obtained on June 30, 2021. The study was approved by the Ethics Committee of the Medical University of Gdańsk, approval number: NKBBN/143/2021. The Ethics Committee waived the requirement for informed consent.

## Results

**Fig 1:**
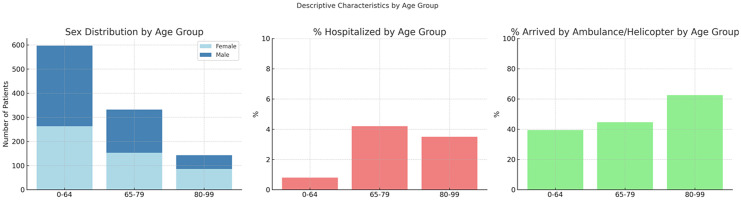
Descriptive characteristics of emergency department patients stratified by age group. **(A)** Sex distribution by age category, shown as a stacked bar plot (Female in light blue, Male in steel blue). **(B)** Proportion of patients hospitalized, including in-hospital deaths. **(C)** Proportion of patients arriving via ambulance or helicopter.

A total of 1,063 patients were included in the study, with a mean age of 63.4 years (interquartile range: Q1 = 44.0, median = 67.0, Q3 = 80.0); among them, 588 were aged 18–64 years (52.9% female), 277 were aged 65–79 years (55.6% female), and 198 were aged ≥80 years (58.6% female). Of the 1,063 patients analyzed, 475 (44.7%) were aged 65 years or older. Descriptive statistics are presented in Fig 1.

The majority of geriatric patients were classified at level 3 in both triage systems: 48.2% in MTS and 82.9% in ESI. Table 1 presents the distribution of triage levels and corresponding hospitalization rates.

**Table 1:** Number and percentage of geriatric patients (aged 65–99) assigned to each triage level in MTS and ESI, with corresponding hospitalization or mortality rates.

Triage Level	MTS – n (%)	Hospitalization – MTS (%)	ESI – n (%)	Hospitalization – ESI (%)
1	7 (1.5%)	28.6	8 (1.7%)	25.0
2	16 (3.4%)	50.0	22 (4.6%)	59.1
3	229 (48.2%)	44.1	394 (82.9%)	41.9
4	209 (44.0%)	39.2	33 (6.9%)	36.4
5	14 (2.9%)	50.0	18 (3.8%)	44.4

**Fig 2:**
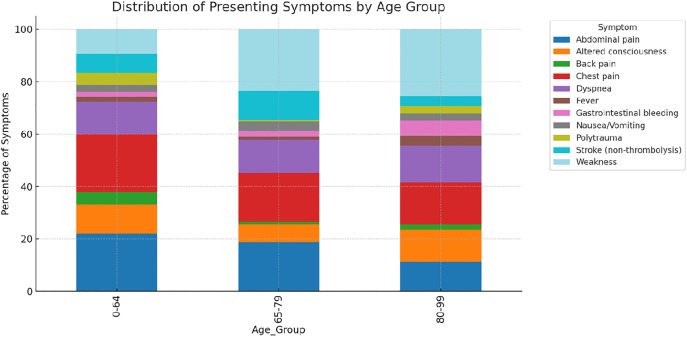
Distribution of presenting symptoms across age groups (18–64, 65–79, 80–99 years; stacked bar chart). Bars sum to 100% within each age category, allowing visual comparison of symptom profiles across age strata.

Presenting symptoms varied significantly across age groups. Among patients aged 18–64 years, abdominal pain and polytrauma were more frequently reported. In contrast, geriatric patients (65–99 years) more commonly presented with dyspnea, weakness, and altered consciousness. The frequency of dyspnea nearly doubled in patients aged ≥80 compared to younger adults, whereas the prevalence of abdominal pain and polytrauma declined with age. Fever and nausea/vomiting were observed at similar rates across all age groups. The detailed distribution of symptoms by age group is shown in Fig 2.

There was no statistically significant association between mode of arrival (EMS/helicopter vs. other) and the risk of hospitalization or in-hospital death (χ² = 0.32, p = 0.571).

**Fig 3:**
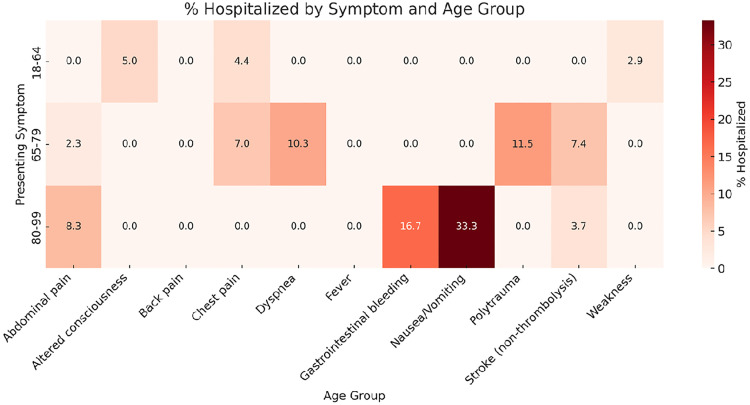
Percentage hospitalized by presenting symptom and age group (heatmap). Cells display within-symptom hospitalization percentages for age strata (18–64, 65–79, 80–99 years); darker shades indicate higher proportions.

The highest hospitalization rates were observed among patients aged 80–99 years presenting with altered consciousness or weakness. Dyspnea was consistently associated with a high hospitalization rate across all elderly subgroups. In contrast, younger adults presenting with abdominal pain or polytrauma had lower hospitalization rates. Detailed percentages are illustrated in Fig 3.

**Fig 4:**
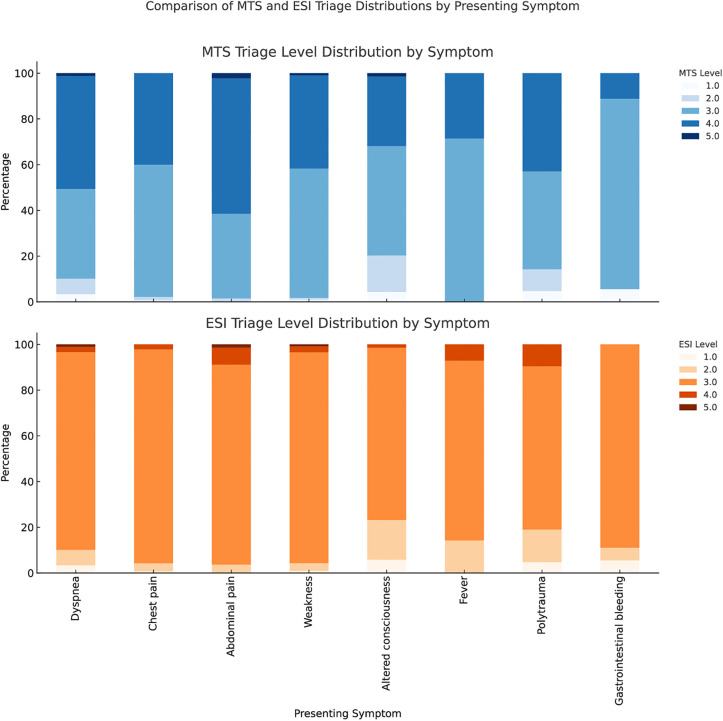
Triage level distributions by presenting symptom for MTS and ESI. For each symptom category, bars show the proportion of patients assigned to levels 1–5 within each triage system, enabling side-by-side comparison of acuity allocation.

The comparison of triage level distributions across both systems based on presenting symptoms is shown in Fig 4. The upper panel displays the proportions of levels in MTS, while the lower panel presents ESI levels for the same symptoms. Overall, the ESI system demonstrated greater sensitivity in differentiating urgent conditions, such as gastrointestinal bleeding and altered consciousness.

**Fig 5:**
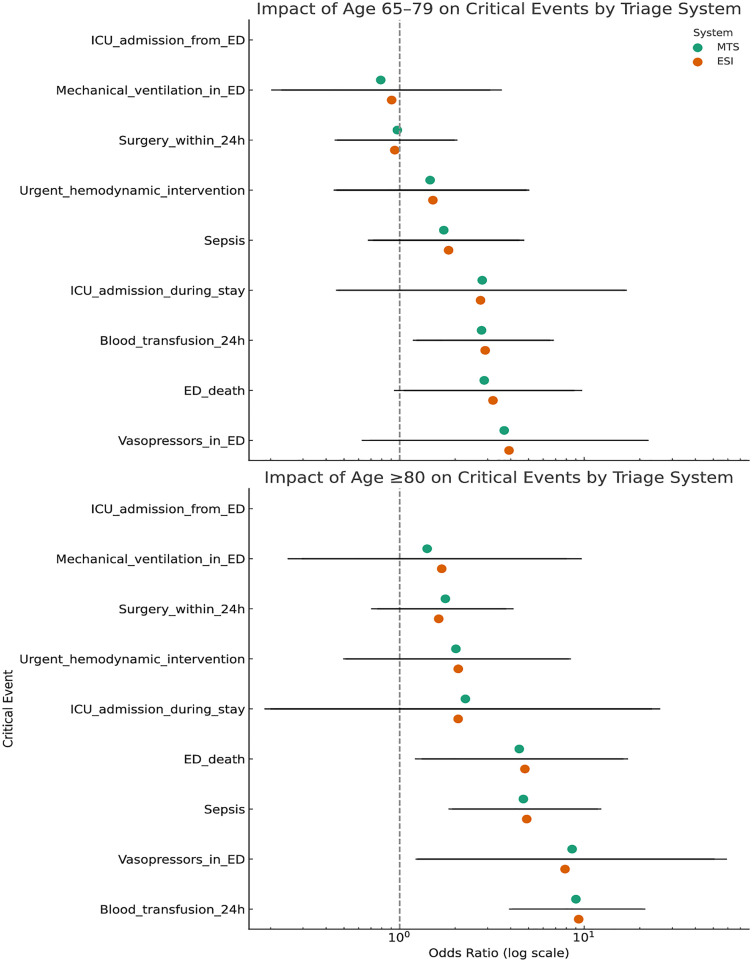
Adjusted odds ratios (OR) for critical events by triage system (MTS vs. ESI) and age group. Points represent ORs with 95% CIs from multivariable models (see Methods for covariates), stratified by age (18–64, 65–79, 80–99). “Critical events” comprise ICU admission (either directly from the ED or during hospitalization), vasopressor use, blood transfusion, or in-hospital death. CI – confidence interval, ICU – intensive care unit.

Logistic regression analysis conducted in the population of patients aged ≥65 years demonstrated that both triage level at admission and advanced age were strongly and independently associated with the risk of critical events. In the model based on MTS, each increase in triage level (i.e., assignment of a lower clinical priority) was associated with a significant 49% reduction in the odds of experiencing a critical event (OR = 0.51; 95% CI: 0.41–0.63; p < 0.001). Age 65–79 years was associated with more than twice the risk (OR = 2.03; p = 0.001), while patients aged ≥80 had nearly a fourfold increase in risk (OR = 3.74; p < 0.001). Similar results were observed for ESI. Each increase in triage level (indicating reduced urgency) was associated with a 57% decrease in the odds of critical events (OR = 0.43; 95% CI: 0.32–0.60; p < 0.001). Older age remained an independent risk factor (OR = 2.16 for ages 65–79 and OR = 3.93 for ≥80 years; both p < 0.001). Further analysis revealed that the oldest age group (particularly ≥80 years) was disproportionately affected by the most clinically significant complications, including sepsis, urgent surgical intervention within 24 hours, ICU admission (either directly from the ED or during hospitalization), and in-hospital mortality. For instance, age ≥ 80 was associated with more than a twofold increase in the risk of ICU transfer during hospitalization (OR = 2.13; 95% CI: 1.36–3.34). The ESI system demonstrated slightly greater consistency in predicting events associated with physiological instability, such as the need for vasopressor use, mechanical ventilation, or sepsis management. A high ESI priority (e.g., level 2 vs. 4/5) was strongly correlated with the occurrence of such critical conditions. Importantly, no significant interaction was observed between triage system and age group, suggesting that the effect of advanced age on critical outcomes operates independently of the assigned clinical priority — see Fig 5. Due to the small number of cases, direct ICU admissions from the ED were combined with ICU admissions during hospitalization into a single outcome category to ensure statistical stability.

## Discussion

This study conducted a comprehensive analysis of patients admitted to the ED, with specific attention to age, mode of arrival, presenting symptoms, triage levels, hospitalization outcomes, and the incidence of critical events. Particular focus was given to the geriatric population (65–99 years), which constituted nearly half of the study sample. The results highlight significant differences in clinical presentation, hospitalization decisions, and outcomes based on age, triage system, and initial complaints.

With increasing age, a marked shift was observed in presenting complaints [12]. While younger patients (<65 years) most frequently reported abdominal pain and trauma, older adults (65–79 and ≥80 years) more commonly presented with dyspnea, weakness, and altered mental status. These findings align with previous studies, which report that approximately 20% of elderly ED patients present with nonspecific symptoms such as weakness, confusion, or dizziness [13]. Among patients ≥80 years, the prevalence of dyspnea nearly doubled compared to younger adults. Such nonspecific complaints, often indicative of serious underlying conditions, may lead to under-triage if not interpreted appropriately in the context of age and physiological reserve [14,15].

Older patients were also more likely to arrive via emergency medical services (EMS) or medical transport [16,17]. However, mode of arrival was not a significant predictor of hospitalization or critical events in multivariable analysis [18]. This may suggest a disconnect between prehospital decision-making and actual clinical risk, or that the ED triage system better captures the patient’s current condition [19].

Both MTS and ESI showed strong associations between assigned triage priority and the risk of hospitalization and critical outcomes. Lower triage levels (higher urgency) were consistently associated with a greater likelihood of complications [7,20]. In regression analysis, each increase in MTS level was associated with a 49% decrease in the odds of a critical event, while ESI showed a 57% reduction. These findings are consistent with other studies [7,21].

Importantly, the ESI system demonstrated greater consistency in classifying patients with severe symptoms, such as altered mental status or gastrointestinal bleeding [22]. This translated into higher predictive values for critical interventions, including sepsis management and mechanical ventilation. In contrast, the symptom-based MTS algorithm displayed greater variability in classification and lower sensitivity, particularly for atypical presentations—a trend also reported in the literature [23,24].

Advanced age was an independent risk factor for most critical events analyzed, including ICU admission, urgent surgery within 24 hours, microbiologically or clinically confirmed sepsis, mechanical ventilation, and in-hospital mortality. Among patients ≥80 years, the risk of ICU admission during hospitalization was more than twice that of younger counterparts, consistent with findings from Australian studies [25].

These results support the growing consensus that existing triage tools, though useful, are not sufficiently adapted to assess risk in older adults—especially those presenting with nonspecific symptoms [26]. The need to modify triage algorithms or incorporate additional tools (e.g., Clinical Frailty Scale, National Early Warning Score 2) has been increasingly emphasized in international literature [27–29]. Our data reinforce this need, showing a systematic underestimation of risk in older adults despite their assigned triage level. An additional noteworthy finding of our study is the markedly high hospitalization rate observed among elderly patients presenting with nausea and vomiting. While this symptom is often perceived as nonspecific, in older adults it may signal acute and severe underlying conditions, including surgical emergencies (e.g., bowel obstruction, mesenteric ischemia), gastrointestinal pathologies (e.g., upper GI bleeding), or neurological disorders (e.g., intracranial hemorrhage, increased intracranial pressure) [30]. This highlights the clinical relevance of seemingly benign complaints in the geriatric population, where atypical or subtle presentations may mask life-threatening illness. Our results therefore emphasize the need for heightened vigilance and a lower threshold for comprehensive evaluation in elderly patients presenting with nausea or vomiting.

## Limitations

This study has several limitations. First, it was retrospective and conducted at a single center, which may limit the generalizability of the findings. Differences in admission thresholds, ICU criteria, and emergency department organization across healthcare systems should be considered when extrapolating these results. Such contextual variability has been shown to influence triage performance and patient outcomes [26].

Second, some potentially relevant variables were unavailable, particularly frailty measures (e.g., Clinical Frailty Scale, functional status, or living conditions) or initial laboratory values. Given the growing evidence that frailty screening impoves risk stratification in geriatric patients with nonspecific presentations, the lack of such data represents an important limitation [27].

Third, although both MTS and ESI were assigned simultaneously, only MTS guided real-time patient management in accordance with hospital policy. ESI levels were recorded for research purposes only, which may have introduced bias, as the two systems did not equally influence downstream care. Similar concerns have been raised in observational validation studies of triage tools [23], highlighting the importance of considering such design-related effects when interpreting comparative performance.

Fourth, the composite endpoint of “critical events” encompassed heterogeneous outcomes (e.g., sepsis, urgent surgery, ICU admission, mechanical ventilation). While this approach ensured sufficient event rates for analysis, it may reduce comparability with other studies. Future research would benefit from standardized outcome frameworks, such as Utstein-style templates for emergency medicine research.

Finally, although our retrospective design precluded calculating classical performance metrics (e.g., sensitivity, specificity, under- or overtriage), the use of objectively defined critical events provided a clinically meaningful and robust endpoint. Prospective multicenter studies remain necessary to validate these findings and assess the adequacy of triage in older adults more comprehensively.

## Conclusions

Our study demonstrates that both MTS and ESI possess limited—yet statistically significant—capacity to predict hospitalization and critical events in geriatric emergency department patients. While both systems effectively stratify clinical risk based on triage levels, ESI showed greater consistency in identifying patients requiring intensive interventions, particularly in cases of physiological deterioration (e.g., mechanical ventilation, vasopressor use, or sepsis).

Advanced age, especially ≥80 years, was a strong independent predictor of hospitalization and serious complications, regardless of the assigned triage level. Furthermore, symptoms commonly seen in older adults—such as weakness, altered mental status, and dyspnea—were significantly associated with higher hospitalization risk, yet may not be adequately weighted in current triage algorithms.

Triage tools designed for the general population may be insufficient for accurately assessing risk in older patients. Our findings support the need to adapt existing systems or implement supplementary indices, such as frailty scores, functional assessments, or geriatric-specific risk stratification models in emergency settings.
